# Dietary protein intake and all-cause mortality: results from The Kawasaki Aging and Wellbeing Project

**DOI:** 10.1186/s12877-023-04173-w

**Published:** 2023-08-09

**Authors:** Hideaki Kurata, Shu Meguro, Yukiko Abe, Takashi Sasaki, Keiko Asakura, Yasumichi Arai, Hiroshi Itoh

**Affiliations:** 1 Division of Endocrinology, Metabolism and Nephrology Department of Internal Medicine, Keio University, School of Medicine, Shinjuku-ku, Tokyo, 160-0016 Japan; 2 Centre for Supercentenarian Medical Research, Keio University School of Medicine, Shinjuku- ku, Tokyo, 160-0016 Japan; 3 Department of Environmental and Occupational Health, School of Medicine, Toho University, Ohta-ku, Tokyo, 143-8540 Japan

**Keywords:** All-cause mortality, Dietary protein intake, Muscle mass

## Abstract

**Background:**

Increased protein intake has been recommended to prevent sarcopenia/frailty, reports on the quantity and quality of protein intake needed and the associated prognosis, particularly in the aging population of Asia, are limited. In this study, we aimed to investigate the relationship between protein intake and mortality in Japanese individuals, aged 85 years and older.

**Methods:**

The data were obtained from The Kawasaki Aging and Wellbeing Project, which is a prospective cohort study of older adults aged between 85 and 89 years with no physical disability at baseline. Of the 1,026 adults in the cohort, 833 were included in the analysis, after excluding those who had not completed a brief, self-administered diet history questionnaire or those who scored less than 24 on the Mini-Mental State Examination. The participants were grouped into quartiles based on protein intake: Q1 (protein < 14.7, %Energy), Q2 (14.7 ≤ protein < 16.7, %Energy), Q3 (16.7 ≤ protein < 19.1, %Energy), and Q4 (≥ 19.1, %Energy). Multivariate Cox proportional hazards models were utilized to evaluate the association between protein intake and all-cause mortality. Kaplan–Meier survival curves were employed to investigate the relationship between protein intake and all-cause mortality.

**Results:**

The mean protein intake of our study population was 17.0% of total energy. Animal protein intake, particularly fish intake, increased significantly along with total protein intake. The study had an average observation period of 1,218 days and recorded 89 deaths. After adjusting for age, sex, skeletal muscle mass index, cardiovascular disease, cancer, education, and serum albumin levels, a lower risk of all-cause mortality was observed in the highest protein intake (Q4) group than in the lowest protein intake (Q1) group (hazard ratio: 0.44, 95% confidence interval: 0.22–0.90, p-value: 0.020).

**Conclusion:**

Protein intake is associated with a reduced risk of all-cause mortality in older adults (aged ≥ 85 years) who engage in independent activities of daily living. This association may impact all-cause mortality independent of muscle mass.

## Background

The relationship between the nutritional composition of the diet and health has been a major medical and frequent research topic [1–4]. In terms of nutrient composition, most attention should be paid to the quality and quantity of protein in particular, as well as the sources of intake. In Europe and the U.S., higher animal protein intake is associated with a higher risk of death from cardiovascular disease (CVD) in the general population [5], whereas higher plant protein intake is associated with a lower risk of death [6, 7]. On the contrary, in China, higher protein intake per se is associated with a lower mortality risk, and no significant association between animal protein intake and mortality risk has been reported [8].

Research findings on the quantity and quality of nutrients in the diet vary widely, which may reflect regional differences in eating habits, as the animal and vegetable proteins consumed also vary. For instance, people in the U.S. consume a higher percentage of fat, whereas people in Japan consume a higher percentage of protein and carbohydrates [9, 10]. Many studies also point to the importance of the source of animal protein. A higher intake of processed and red meat has been reported to increase the risk of death [11], whereas fish consumption has been reported to contribute to a lower risk of mortality [12, 13]. In Western countries, a high percentage of animal protein is derived from red meat and processed meat [6, 7], whereas in Japan, most animal protein is derived from fish and shellfish [6]. In assessing the impact of protein intake and quality on health, it is thus essential to identify regional differences in food culture and examine the data carefully.

Age should also be considered when it comes to food and health issues. Extending healthy life expectancy and improving the quality of life in an aging society is a challenge for countries worldwide. Frailty, which impairs activities of daily living (ADL) in older people [14], is one of the risk factors for their mortality [15]. Several reports have shown that the higher the protein intake, the lower the incidence of frailty [16, 17]; therefore, aggressive protein intake has been advocated for older people [18]. On the contrary, approximately 95% of older people in Japan do not experience frailty until age 75 years [19]. However, few large-scale studies have been conducted on protein intake and prognosis in adults aged ≥ 75 years. In one of the few reports on this topic, Mendonca et al. found that a higher protein intake, particularly when combined with greater physical activity, may delay the incidence of disability in very old adults in a study of Caucasian individuals aged ≥ 85 years [20].

Thus, although the dangers of frailty and the importance of its prevention have been emphasized, data showing how increased protein intake improves mortality in the generation with the highest incidence of frailty remain insufficient. Moreover, protein intake is often discussed in the context of sarcopenia prevention, but whether the prognostic impact of protein intake can be defined by muscle mass alone remains unclear. In particular, large-scale studies on protein intake in older persons aged ≥ 75 years with preserved ADL are lacking. Thus, the prognostic impact of increased protein intake remains unclear.

In the light of this, this study aimed to investigate the relationship between protein intake and mortality in Japanese adults aged ≥ 85 years.

## Materials and methods

### Study population

The Kawasaki Aging and Wellbeing Project (KAWP) is a prospective cohort study of older adults aged between 85 and 89 years, with no physical disability at baseline. The main purpose of the KAWP is to explore trajectories of functional decline, frailty, and cognitive impairment, and to identify genetic, biological, behavioral, and socioenvironmental factors that delay or modify this deteriorating process at an advanced age. The inclusion criteria of KAWP are as follows: (1) being a resident of Kawasaki city, a city with a population of 1.5 million, located in the greater Tokyo area and aged between 85 and 89 years; (2) having no limitations in performing basic ADLs; and (3) being able to visit the study site, the Kawasaki Municipal Hospitals, independently. As of March 2017, 12,906 individuals were screened as potential participants, using the basic registration of residents and the long-term care insurance database. Among them, we sent an invitation to participate in this study to 9,976 individuals, and 1,464 eligible residents responded to express their willingness to participate in the study. Between March 2017 and December 2018, a total of 1,026 independent older people were enrolled in the KAWP. A comprehensive baseline assessment, including assessment of physical, mental, and cognitive function, as well as social participation, was conducted. Specifically, the following tests were performed at three hospitals in Kawasaki City at the site of the basic survey: medical examination (interview, blood pressure measurement, and consultation), physical fitness test (grip strength, walking speed, and lower-limb muscle strength), carotid artery echocardiogram, hearing test, cognitive function test, measurement of muscle mass by bioimpedance method, blood test, urine test, bone density test, and spinal X-ray. Several papers on the KAWP cohort have already been published [21–23]. The participants were scheduled for telephonic surveys every 6 months to monitor their vital status [24], any incidental disabilities, trauma from falls and fractures, and hospitalizations until December 2024, or until they dropped out of the study. Three and 6 years after the start of the study, a survey was planned to include medical examination, physical fitness tests, and echocardiography. However, due to the spread of the new coronavirus infection, a 3-year follow-up survey scheduled for 2020 was postponed; after a delay of 1.5 years, it is now underway.

Written informed consent to take part in the KAWP was obtained from all participants. The KAWP was approved by the ethics committee of the Keio University School of Medicine (ID: 20,160,297) and was registered in the University Hospital Medical Information Network Clinical Trial Registry as an observational study (ID: UMIN000026053).

Eleven participants who filled out the self-administered brief diet history questionnaire (BDHQ) insufficiently and 15 participants with daily energy intake outside the range of 400–6000 kcal inferred from their completed BDHQ were excluded. In a validation study of the BDHQ in older adults aged ≥ 80 years [25], the mean Mini Mental State Examination (MMSE) of the target population was 27.7. In order to ensure the accuracy of the BDHQ in our study, we excluded participants with an MMSE score < 24. Finally, 833 participants were included in our analysis.

### Diet history questionnaire

Nutrient and food intake was estimated using the BDHQ, which consisted of four pages with 90 items addressing foods and dietary habits of the previous month and takes approximately 15–20 min to complete. The BDHQ consists of five sections: (i) frequency of food and non-alcoholic beverage intake, (ii) daily intake of rice and miso soup, (iii) frequency and amount per serving of alcoholic beverage consumption, (iv) usual cooking methods, and (v) general eating behavior. The foods and beverages included in the BDHQ were selected primarily from the food lists used in the Japanese National Health and Nutrition Survey [26], which are commonly consumed in Japan. Standard portion sizes and the sizes of bowls for rice and cups for miso soup were derived from several recipe books for Japanese dishes [27, 28]. Major sources of protein include the following foods: low-fat milk, full-cream milk, chicken, pork, beef, ham, sausage, bacon, liver, squid, shrimp, octopus, shellfish, fish eaten with the bones, canned tuna, dried fish, fatty fish, less fatty fish, eggs, tofu and thick fried tofu, and natto. The BDHQ provides insight into the habitual nutrient intake, which is the intake of about 30 nutrients and 50 foods that people living in Japan consume from their normal diet. The validity of the BDHQ for Japanese older people aged ≥ 80 years has been demonstrated in previous studies [25]. Our research group has already reported a cross-sectional analysis using BDHQ data for the KAWP cohort [24].

To analyze nutrient data, the density method was used to estimate intake per 1,000 kcal. The following formula was used to calculate the energy intake ratio [%Energy (%E)] of energy-producing nutrients (proteins, lipids, and carbohydrates): energy intake from each nutrient / energy intake × 100, since the Dietary Reference Intakes in Japan, the U.S., and other countries use the %E value as the reference value.

### Baseline characteristics

Sociodemographic and health data were obtained from the KAWP baseline survey conducted between March 2017 and December 2018. All participants were invited to visit one of three Hospitals in Kawasaki (Kawasaki, Ida, or Tama) and were interviewed and examined using a study protocol that was harmonized with the Tokyo Oldest Old Survey on Total Health and the Japan Semi-Supercentenarian Study, both of which were managed by the Center for Super-Centenarian Medical Research, Keio University School of Medicine [29, 30]. Participants were asked to fill out a pre-mailed questionnaire that included questions about education (whether or not they were college graduates), living situation, smoking status, the number of remaining teeth, and self-rated health. Their responses were confirmed for consistency by interviewers at the time of the baseline survey. The medical interview was conducted by trained physicians, and the number of chronic conditions was counted based on previous and current medical history of 18 diseases: cerebrovascular disease, cardiac disease, hypertension, diabetes, dyslipidemia, respiratory disease, gastrointestinal disease, renal disease, prostate disease, thyroid disease, Parkinson’s disease, connective tissue disease, eye disease, osteoporosis, arthritis, hyperuricemia, cancer, and dementia. CVD was recalculated as a combination of cardiac disease and cerebrovascular disease. The BDHQ survey was conducted by several trained and experienced staff.

ADLs were checked by use of the Barthel Index (0–100 points) [31], instrumental ADL (IADL) were assessed using the Lawton scale (0–5 points) [32], and cognitive functions were evaluated according to the MMSE (0–30 points) [33]. Blood and urine samples were taken at the three aforementioned hospitals, the sites of the basic survey. Blood tests measured serum albumin, triglycerides, low-density lipoprotein, high-density lipoprotein, C-reactive protein, hemoglobin A1c, urea nitrogen, creatinine, cystatin. Urinalysis measured urinary albumin creatinine ratio. Body composition, specifically, visceral fat area, percentage of body fat, and skeletal muscle index, was measured by the impedance method, using the Body Composition Analyzer InBody S10 (InBody Japan Inc, Tokyo, Japan).

### Mortality assessment

Mortality information was obtained from the long-term care insurance system, which is a nationwide comprehensive welfare insurance scheme covering most of the care services for older people [34]. The plan was to collect data every 6 months and tabulate the data at the 3-year point. However, due to the outbreak of the coronavirus disease 2019 pandemic, this was delayed by 1.5 years. Therefore, we analyzed mortality data through December 2021.

### Statistical analysis

Participants were grouped according to their protein intake quartiles: Q1 (protein < 14.7, %E), Q2 (14.7 ≤ protein < 16.7, %E), Q3 (16.7 ≤ protein < 19.1, %E), and Q4 (≥ 19.1, %E). Baseline characteristics were expressed as mean and standard deviation; categorical variables were shown as numbers and proportions. Skeletal muscle mass index (SMI) is sex-disaggregated; thus, results are also listed by sex. A trend test was performed for continuous variables, and a chi-square test was performed for categorical variables. Association trends between quartile groups of protein intake and other continuous variables were tested using a linear regression model that assigned scores to the independent variable levels (i.e., Q1 of protein intake = 1, Q2 = 2, and so on). Cumulative Kaplan–Meier curves were plotted to depict the relationship between protein quartile groups, and log-rank tests were conducted to assess differences in survival among the groups. The intake of protein, fish, n-3-polyunsaturated fatty acids (PUFAs), and other nutrients were divided into quartiles, and Cox proportional hazards regression analysis was employed to estimate the hazard ratio (HR) and 95% confidence interval (CI) for all-cause mortality in each group, with reference to the group with the lowest protein intake [35]. Age, sex, SMI, education, CVD, cancer, and serum albumin levels were included as covariates in the Cox regression analysis, as these factors may impact prognosis, standard of living, and protein intake in older individuals. Furthermore, a trend test was performed to examine the risk of all-cause mortality across each quartile. Association trends in each quartile group were tested using Cox regression analysis that assigned scores to the independent variable levels (i.e., Q1 of protein intake = 1, Q2 = 2, and so on).

All analyses were performed using SPSS Statistics ver. 29.0 software (IBM SPSS Inc., Armonk, NY, USA). Results were considered statistically significant at a P-value of < 0.05, and two-sided tests were applied.

## Results

The baseline characteristics according to quartiles of protein intake are shown in Table 1. The average age of our study participants was 86.5 ± 1.36 years, the proportion of women was 50.6%, and the average body mass index (BMI) was 23.1 kg/m^2^. The mean values of the Barthel Index, a measure of the ADLs, and the Lawton scale, a measure of the IADLs, fell within the normal score limits. Mean serum albumin was 4.16 mg/dl, mean estimated glomerular filtration rate (eGFR) based on creatinine was 59.3 ml/min/1.73 m^2^, and the mean eGFR based on cystatin was 57.3 ml/min/1.73 m^2^. Mean triglycerides, high-density lipoprotein, C-reactive protein, hemoglobin A1c, and urea nitrogen levels were within the normal range. The mean SMI was 7.33 kg/m^2^, and was 8.25 kg/m^2^ and 6.45 kg/m^2^ for men and women, respectively. In addition, the proportion of females was higher among those with a higher protein intake, while those with a higher protein intake also had a higher number of remaining teeth. Muscle mass displayed the opposite trends. However, when analyzed by sex, no trend association was found between SMI and protein intake. No significant associations were found between protein intake and albuminuria, urea nitrogen levels, a history of cancer or CVD, or an educational background. Mean LDL was mildly elevated in general, but showed no significant trend association with protein intake.

**Table 1:** Baseline characteristics by quartiles of protein intake (N = 833)

Characteristics	mean/n	SD/%	Protein intake (%Energy)				
			Q1 –14.7 N = 208	Q2 –16.7 N = 208	Q3 –19.1 N = 209	Q4 19.1– N = 208	P value
Age (years)	86.5	1.36	86.6	86.5	86.5	86.3	0.042
Sex (female)	422	50.6	37.9	51.4	53.1	60.0	0.002
BMI (kg/m^2^)	23.1	3.16	23.3	22.9	23.2	22.9	0.49
Cancer (n)	182	22.0	48	46	38	50	0.77
CVD (n)	268	32.1	70	66	63	69	0.87
Residual teeth number	13.8	9.85	12.6	12.9	14.6	14.9	0.005
MMSE score	26.9	1.93	26.8	26.9	27.0	26.9	0.37
Barthel Index score	98.5	3.49	98.4	98.6	98.6	98.5	0.63
Lawton Scale score	4.87	0.39	4.89	4.84	4.89	4.88	0.65
Secondary education attainment (n)	271	32.6	69	69	70	63	0.63
Serum Albumin (mg/dl)	4.16	0.28	4.16	4.11	4.17	4.19	0.056
TG (mg/dl)	130	71.2	126	137	130	128	0.92
LDL (mg/dl)	110	27.5	110	109	109	114	0.09
HDL (mg/dl)	61.2	16.0	60.5	60.8	60.9	62.6	0.22
CRP (mg/dl)	0.21	0.57	0.19	0.24	0.19	0.22	0.17
HbA1c (%)	5.97	0.58	5.88	5.96	6.01	6.02	0.004
UN (mg/dl)	19.9	5.73	18.7	19.8	20.6	20.6	0.11
eGFR Cre (ml/min/1.73 m^2^)	59.3	14.5	59.6	59.4	57.9	60.2	0.86
eGFR Cys (ml/min/1.73 m^2^)	57.3	13.9	56.9	56.0	56.8	59.4	0.07
Albuminuria (n)	102	12.3	28	24	23	27	0.89
VFA (cm^2^)	63.6	26.7	64.0	63.2	63.9	63.5	0.98
PBF (%)	27.8	8.04	27.5	27.7	28.0	28.2	0.28
SMI (kg/m^2^) Male Female	7.33 8.25 6.45	1.30 0.05 0.03	7.54 8.23 6.46	7.29 8.29 6.42	7.32 8.12 6.45	7.19 8.16 6.49	0.018 0.79 0.31

P value was assessed with trend test or chi-square tests on mean values of each quartile

Abbreviations: BMI: Body Mass Index, CVD: cardiovascular disease, MMSE: Mini Mental State Examination, TG: triglycerides, LDL: low density lipoprotein, HDL: high density lipoprotein, CRP: C-reactive protein, HbA1c: hemoglobin A1c, UN: urea nitrogen, eGFR: estimated glomerular filtration rate, Cre: creatinine, Cys: cystatin, VFA: visceral fat area, PBF: percentage of body fat, SMI: skeletal muscle index

The BDHQ results according to quartile of protein intake are shown in Table 2. The average protein intake was 17.0%E. The mean protein intake for the first quartile (Q1) was 13.1%E, Q2 was 15.7%E, Q3 was 17.9%E, and Q4 was 21.2%E. The average daily energy intake for each quartile group was approximately 2,000 kcal. As protein intake increased, carbohydrate intake decreased and fat intake increased. Moreover, as protein intake increased, plant protein intake remained constant, but animal protein intake increased. Animal proteins were obtained primarily from fish and meat. In particular, the protein intake from fish was approximately 3.5 times as high in the fourth than in the first quartile.

**Table 2:** BDHQ results by quartiles of protein intake (N = 833)

Characteristics	mean/n	SD/%	Protein intake (%Energy)				
			Q1 –14.7 N = 208	Q2 –16.7 N = 208	Q3 –19.1 N = 209	Q4 19.1– N = 208	P value
Energy (kcal/day)	2,038	606	1,957	2,041	2,080	2,076	0.016
Carbohydrate (%E)	50.5	7.64	55.4	53.3	49.1	44.1	<0.001
Protein (%E)	17.0	3.18	13.1	15.7	17.9	21.2	<0.001
Animal protein (%E)	10.3	3.35	6.52	9.06	11.1	14.5	<0.001
Plant protein (%E)	6.71	1.15	6.66	6.71	6.75	6.72	0.58
Fish (g/1,000 kcal)	42.2	25.5	20.3	34.3	45.6	68.6	<0.001
Meats (g/1,000 kcal)	45.8	29.8	29.0	40.1	51.3	62.4	<0.001
Eggs (g/1,000 kcal)	27.8	17.2	19.5	26.8	31.6	33.4	<0.001
Dairy products (g/1,000 kcal)	108	67.0	90.1	111	110	121	<0.001
Fat (%E)	29.4	5.29	25.9	28.1	30.9	32.8	<0.001
Total fatty acid (%E)	25.4	4.66	22.5	24.2	26.6	28.0	<0.001
SFA (%E)	8.10	1.84	7.40	7.87	8.34	8.77	<0.001
MUFA (%E)	10.3	2.14	9.18	9.88	10.9	11.4	<0.001
PUFA (%E)	6.92	1.44	5.96	6.52	7.35	7.82	<0.001
n-6PUFA (%E)	5.39	1.16	4.82	5.14	5.72	5.85	<0.001
n-3PUFA (%E)	1.50	0.44	1.11	1.35	1.60	1.92	<0.001
EPA (%E)	0.20	0.12	0.10	0.16	0.21	0.31	<0.001
DPA (%E)	0.57	0.30	0.32	0.49	0.61	0.86	<0.001
DHA (%E)	0.33	0.01	0.18	0.28	0.35	0.50	<0.001

P-value was assessed with trend test on mean values of each quartile

Abbreviations: SFA: saturated fat acids, MUFA: monounsaturated fat acids, PUFA: polyunsaturated fat acid, EPA: eicosapentaenoic acid, DPA: docosapentaenoic acid, DHA: docosahexaenoic acid

**Fig. 1:**
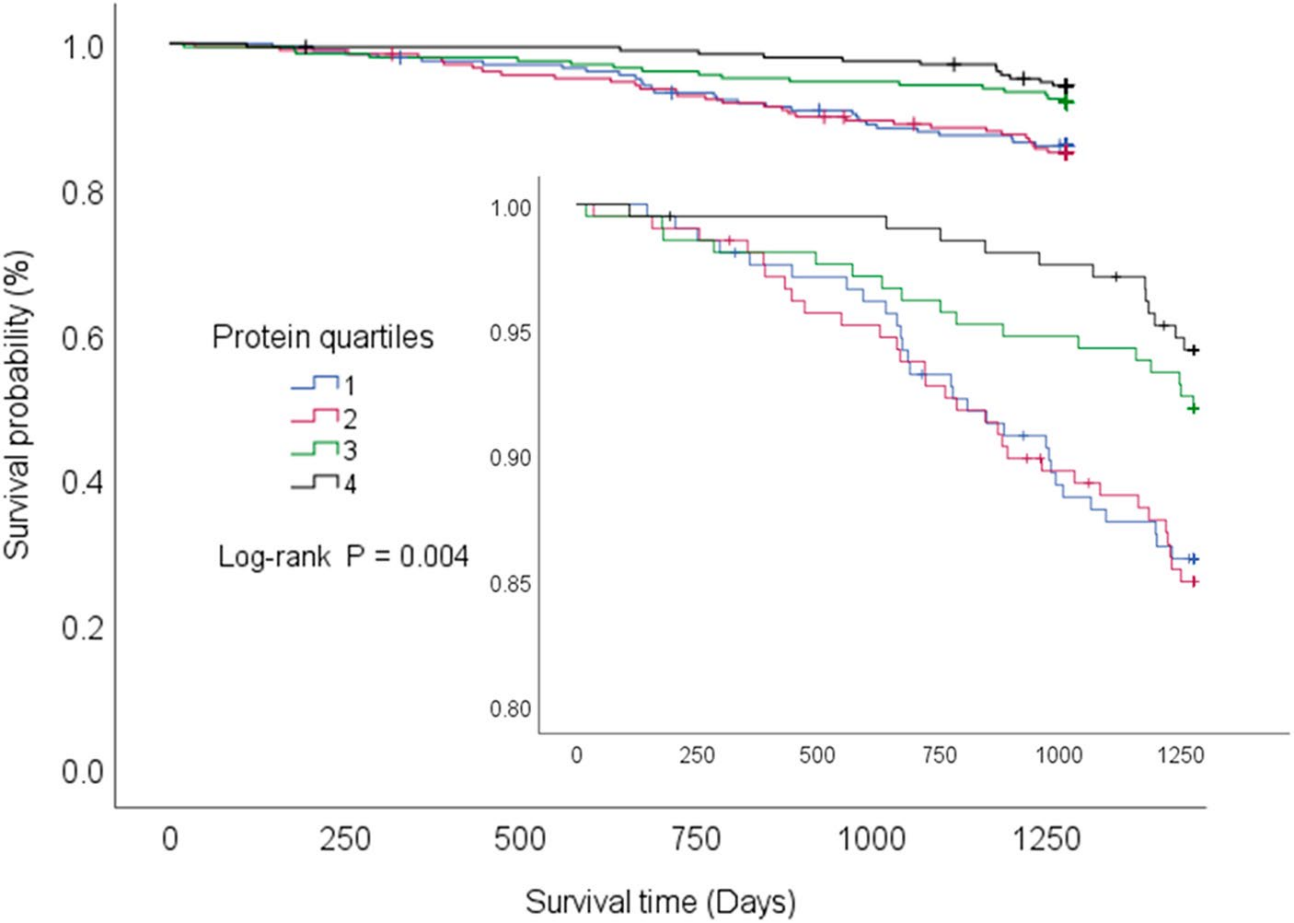
Cumulative survival rate and categorical protein intake vs. follow up duration

As shown in Fig. 1, during the 3.5-year follow-up, with an average observation period of 1,218 days, 29 deaths occurred in group Q1, 31 in group Q2, 17 in group Q3, and 12 in group Q4. Survival time analysis for groups Q1–4 showed significantly longer survival in the high protein intake group.

HRs and 95% CIs for the contribution of quartiles of protein per se, animal protein, protein-rich food, plant protein, and n-3PUFA intake to death over a 3.5-year period are shown in Table 3. To determine the relationship between the intake of these foods and all-cause mortality, we performed an analysis using a Cox regression model. The Q4 group, which had the highest protein intake, had a significantly lower HR (0.44 [95% CI, 0.22–0.90]; P = 0.020) than did the Q1 group. Similarly, for fish intake, the Q4 group had a lower HR than did the Q1 group. The trends for protein intake were significant. When BMI was used as a covariate instead of SMI, the results were consistent with those presented in Table 3.

**Table 3:** Hazard ratios and 95% confidence intervals for the contribution of quartiles of protein, animal protein, protein-rich food, plant protein, and n-3PUFA intake to death over a 3.5-year period

	Q1	Q2		Q3		Q4		P trend
		HR	95%CI	HR	95%CI	HR	95%CI	
Model 1								
Protein (%E)	ref	1.15	0.69–1.94	0.63	0.35–1.16	0.45	0.22–0.88	0.006
Animal protein (%E)	ref	1.09	0.63–1.88	0.93	0.53–1.64	0.53	0.27–1.03	0.06
Plant protein (%E)	ref	0.85	0.48–1.49	0.79	0.43–1.42	0.61	0.33–1.14	0.12
Fish (g/1,000 kcal)	ref	0.89	0.50–1.58	1.04	0.60–1.80	0.46	0.22–0.93	0.08
Meats (g/1,000 kcal)	ref	0.69	0.38–1.26	0.76	0.42–1.38	0.83	0.46–1.50	0.57
Eggs (g/1,000 kcal)	ref	1.12	0.63–2.00	1.18	0.67–2.07	0.67	0.33–1.37	0.48
Dairy products (g/1,000 kcal)	ref	1.05	0.60–1.82	0.77	0.41–1.41	0.55	0.28–1.11	0.08
n-3PUFA (%E)	ref	0.77	0.44–1.35	0.68	0.38–1.22	0.53	0.28–1.00	0.043
Model 2								
Protein (%E)	ref	1.11	0.66–1.88	0.67	0.36–1.22	0.44	0.22–0.90	0.010
Animal protein (%E)	ref	1.05	0.60–1.83	1.03	0.58–1.83	0.53	0.26–1.05	0.11
Plant protein (%E)	ref	0.81	0.46–1.42	0.75	0.41–1.36	0.58	0.33–1.07	0.08
Fish (g/1,000 kcal)	ref	0.89	0.51–1.58	1.14	0.65–1.98	0.48	0.23–0.97	0.13
Meats (g/1,000 kcal)	ref	0.67	0.37–1.21	0.70	0.38–1.28	0.82	0.45–1.49	0.51
Eggs (g/1,000 kcal)	ref	1.19	0.67–2.12	1.22	0.69–2.14	0.69	0.34–1.41	0.52
Dairy products (g/1,000 kcal)	ref	1.01	0.58–1.76	0.77	0.42–1.42	0.55	0.28–1.10	0.07
n-3PUFA (%E)	ref	0.82	0.47–1.44	0.72	0.36–1.30	0.56	0.26–1.06	0.08

Covariates: Model 1 included age, sex, SMI, secondary education, cancer, and CVD; Model 2 included Model 1 plus serum albumin levels

P-value for the trends of association among each quartile group of protein intake or other continuous variables

Abbreviations: HR: hazard ratio, CI: confidence interval

## Discussion

Our study found that older individuals aged ≥ 85 years with independent ADLs who consumed higher amounts of protein had a lower risk of mortality. As an observational study, it is difficult to establish a causal relationship between protein intake and mortality based solely on our results. However, our findings suggest that protein intake may play a significant role in the all-cause mortality of a large number of older individuals, even after adjusting for as many confounding factors as possible (Table 3). The blood and urine test results for all of our study participants were mostly within the normal range, with no clinically problematic values. The results for the Q4 group showed no significant increase in the prevalence of albuminuria, urea nitrogen, or serum creatinine levels, and no negative effects of a high protein intake (Table 1). Although the target protein intake in Japan is 15–20%E [36], the upper limit of the protein intake standard in Japan has been established based on reports from a review of mainly Caucasians who were in their sixties [37]. This means that the race, age, and eating habits of the target population were not taken into account, and the adaptation of these findings to Japanese people, particularly older people, can be debated. Our study included only the very old Japanese population. We believe we have reported valuable results on nutrition and mortality related to older people in Asia.

The results of this study suggested two mechanisms by which protein intake affects mortality. The first is fish consumption. Fish is rich in n-3 unsaturated fatty acids and provides a balance between high-quality protein and fat. In our study population, as protein intake increased, the proportion of animal protein intake, particularly fish intake, became significantly larger (Table 2), unlike previous reports from Western countries [6, 7]. The Q4 group, which had the highest intake of fish, had a significantly lower all-cause mortality rate than did the Q1 group (Table 3). Fish contain good fats, such as n-3 unsaturated fatty acids, along with abundant nutrients, such as vitamins [38, 39], essential amino acids [40, 41], and trace elements [42, 43]. In addition, n-3 unsaturated fatty acids have been reported to have anti-inflammatory effects [44], inhibit carcinogenesis of some cancer types [45–50], and prevent the onset of coronary artery disease [51]. In addition, a high intake of fish leads to a relative reduction in the intake of saturated fatty acids, which are abundant in animal proteins, particularly meat. Saturated fatty acids are a risk factor for CVD-related mortality [52], and their excessive intake should be avoided. Thus, the abundant nutrients in fish, the multifaceted effects of n-3PUFAs present in fish, and the improved fatty acid intake balance associated with fish intake may contribute to reduced mortality.

The other is the maintenance and improvement of nutritional status. Albumin, the major component of serum protein, is synthesized in the liver and reflects the nutritional status of the body. A positive association between serum albumin and animal protein intake has been reported [53], and hypoalbuminemia has been reported to be a risk factor for all-cause mortality and CVD [54–56]. Our study confirmed a positive trend in protein intake and serum albumin levels (Table 1). This result may suggest that good nutritional status due to protein intake is one factor that reduces mortality rates. However, the effect of protein and fish consumption on mortality is multifactorial and cannot be attributed to a single factor. In the model adjusted for serum albumin levels and other factors, unlike n-3PUFAs, fish intake equivalent to Q4 levels also contributed to mortality reduction (Table 3). This may reflect the fact that the intake of abundant nutrients other than n-3PUFA contributed to the reduction in mortality through a mechanism independent of serum albumin.

Our analysis found that the beneficial effect of protein intake on mortality was independent of muscle mass. This result may be because our study included only participants who maintained their ADL, indicating that other factors may also contribute to the observed association.

Our study included the following limitations: First, no cutoff value for protein was set for clinical use: although Q4 intake (≥ 19.1, %E) was associated with a lower mortality than Q1, that cutoff value differed from the clinically prevalent value. Our study used quartiles as cutoffs and did not take ease of use into account. Second, the causes of death were unknown. Animal protein intake has been reported to be a risk factor for CVD [5]. Although associations between high protein intake and the risk of mortality in Asian individuals have rarely been reported, we cannot rule out the possibility that cause-of-death bias may underestimate the negative effects of meat-derived proteins, such as those obtained from processed meat. Third, the follow-up period was shorter than that of other studies, with fewer mortality events. We cannot rule out the possibility that longer-term follow-up would change the results. Fourth, reverse causality may be possible. The study was conducted among older people aged ≥ 85 years, and it is undeniable that those who can consume more protein are healthier and physically stronger. The fifth is the loss of power due to protein categorization for analysis. The sixth is external and internal validity. The KAWP participants are urban, socioeconomically advantaged, older Asian adults with a high fish intake. Moreover, they have no organ damage or reduced ADLs. Therefore, generalizability should be interpreted in the light of the particularities of the participants in this study. The number of death events over the 3.5-year period was limited and did not allow for inclusion of a sufficient number of covariates in the Cox analysis. Although the analysis was performed using as many covariates as possible, it was not free from confounding effects. However, the protocol of our study takes into account the lack of follow-up, and telephonic surveys were conducted every 6 months. During the interviews, several experienced and trained staff members assisted with the questionnaires. Information on deaths was based on long-term care insurance and was highly accurate and reliable.

The strengths of our study were two-fold. First, we were able to analyze the dietary habits and mortality of approximately 850 older individuals aged ≥ 85 years with independence in ADLs. The participants were independent in their daily lives, indicating that this allowed us to analyze nutritional status and mortality after excluding one factor that has a significant impact on prognosis. Notably, large-scale studies on older individuals aged ≥ 85 years are rare, and therefore, valuable. Second, the analysis considered various factors, including blood markers, urinalysis, body composition, and social background. We measured muscle mass, which can influence survival in older people, and adjusted for this to determine the prognostic impact (Table 3). Protein intake limits are often implemented due to concerns about the burden on renal function. However, in our study, eGFR showed no inverse association with protein intake (Table 1), which may indicate that it is not necessary for older adults in good health to restrict protein intake, and, in fact, that higher protein intake had positive health associations.

## Conclusion

This study, based on the KAWP, revealed that protein intake is associated with a reduced risk of all-cause mortality in older adults aged ≥ 85 years with independence in ADLs. This association may contribute to improved mortality, independent of muscle mass. Greater emphasis on increased protein intake is required to improve the health of older Asian individuals.

## Data Availability

The data will be made available upon request with an appropriate research arrangement with approval of the Research Ethics Committee of Keio University School of Medicine for Clinical Research. Thus, to request the data, please contact Dr. Yasumichi Arai (PI of the KAWP) via e-mail: yasumich@keio.jp.

## Abbreviations

%E: %Energy
ADL: Activities of daily living
BDHQ: Brief-type self-administered diet history questionnaire
BMI: Body mass index
CI: Confidence interval
Cre: Creatinine
CRP: C-reactive protein
CVD: Cardiovascular disease
Cys: Cystatin
DHA: Docosahexaenoic acid
DPA: Docosapentaenoic acid
eGFR: Estimated glomerular filtration rate
EPA: Eicosapentaenoic acid
HbA1c: Hemoglobin A1c
HDL: High density lipoprotein
HR: Hazard ratio
IADL: Instrumental activities of daily living
KAWP: The Kawasaki Aging and Wellbeing Project
LDL: Low density lipoprotein
MMSE: Mini-Mental State Examination
MUFA: Monounsaturated fatty acid
PBF: Percentage of body fat
PUFA: Polyunsaturated fatty acid
SD: Standard deviation
SFA: Saturated fatty acid
SMI: Skeletal muscle mass index
TG: Triglycerides
UN: Urea nitrogen
VFA: Visceral fat area

## References

[1] Sofi F, Cesari F, Abbate R, Gensini GF, Casini A (2008). Adherence to Mediterranean diet and health status: meta-analysis. BMJ.

[2] Oussalah A, Levy J, Berthezène C, Alpers DH, Guéant J-L (2020). Health outcomes associated with vegetarian diets: an umbrella review of systematic reviews and meta-analyses. Clin Nutr.

[3] Bakaloudi DR, Halloran A, Rippin HL, Oikonomidou AC, Dardavesis TI, Williams J (2021). Intake and adequacy of the vegan diet. A systematic review of the evidence. Clin Nutr.

[4] Paoli A, Rubini A, Volek JS, Grimaldi KA (2013). Beyond weight loss: a review of the therapeutic uses of very-low-carbohydrate (ketogenic) diets. Eur J Clin Nutr.

[5] Chen Z, Glisic M, Song M, Aliahmad HA, Zhang X, Moumdjian AC (2020). Dietary protein intake and all-cause and cause-specific mortality: results from the Rotterdam Study and a meta-analysis of prospective cohort studies. Eur J Epidemiol.

[6] Budhathoki S, Sawada N, Iwasaki M, Yamaji T, Goto A, Kotemori A (2019). Association of animal and plant protein intake with all-cause and cause-specific mortality in a japanese cohort. JAMA Intern Med.

[7] Song M, Fung TT, Hu FB, Willett WC, Longo VD, Chan AT, Giovannucci EL (2016). Association of animal and plant protein intake with all-cause and cause-specific mortality. JAMA Intern Med.

[8] Chan R, Leung J, Woo J (2019). High protein intake is associated with lower risk of all-cause mortality in community-dwelling chinese older men and women. J Nutr Health Aging.

[9] U.S. Department of Agriculture, Agricultural Research Service. 2020. Nutrient Intakes from Food and Beverages: Mean Amounts Consumed per Individual, by Gender and Age, What We Eat in America, NHANES 2017–2018.

[10] 2019 National Health and Nutrition Survey Report. Part 1: Results of the nutrient intake survey (in Japanese). https://www.mhlw.go.jp/content/000711006.pdf. Accessed 20 Mar 2023.

[11] Schwingshackl L, Schwedhelm C, Hoffmann G, Lampousi A-M, Knüppel S, Iqbal K (2017). Food groups and risk of all-cause mortality: a systematic review and meta-analysis of prospective studies. Am J Clin Nutr.

[12] Zhao LG, Sun JW, Yang Y, Ma X, Wang YY, Xiang YB (2016). Fish consumption and all-cause mortality: a meta-analysis of cohort studies. Eur J Clin Nutr.

[13] Yamagishi K, Iso H, Date C, Fukui M, Wakai K, Kikuchi S (2008). Fish, omega-3 polyunsaturated fatty acids, and mortality from cardiovascular diseases in a nationwide community-based cohort of japanese men and women the JACC (Japan Collaborative Cohort Study for evaluation of Cancer Risk) Study. J Am Coll Cardiol.

[14] Xue QL, Bandeen-Roche K, Varadhan R, Zhou J, Fried LP (2008). Initial manifestations of frailty criteria and the development of frailty phenotype in the women’s Health and Aging Study II. J Gerontol A Biol Sci Med Sci.

[15] Hanlon P, Nicholl BI, Jani BD, Lee D, McQueenie R, Mair FS (2018). Frailty and pre-frailty in middle-aged and older adults and its association with multimorbidity and mortality: a prospective analysis of 493 737 UK Biobank participants. Lancet Public Health.

[16] Coelho-Júnior HJ, Rodrigues B, Uchida M, Marzetti E (2018). Low protein intake is associated with frailty in older adults: a systematic review and meta-analysis of observational studies. Nutrients.

[17] Lorenzo-López L, Maseda A, de Labra C, Regueiro-Folgueira L, Rodríguez-Villamil JL, Millán-Calenti JC (2017). Nutritional determinants of frailty in older adults: a systematic review. BMC Geriatr.

[18] Coelho-Junior HJ, Marzetti E, Picca A, Cesari M, Uchida MC, Calvani R (2020). Protein intake and frailty: A matter of quantity, quality, and timing. Nutrients.

[19] Taniguchi Y, Kitamura A, Nofuji Y, Ishizaki T, Seino S, Yokoyama Y (2019). Association of trajectories of higher-level functional capacity with mortality and medical and long-term care costs among community-dwelling older japanese. J Gerontol A Biol Sci Med Sci.

[20] Mendonça N, Kingston A, Granic A, Hill TR, Mathers JC, Jagger C (2020). Contribution of protein intake and its interaction with physical activity to transitions between disability states and to death in very old adults: the Newcastle 85 + study. Eur J Nutr.

[21] Ando T, Nishimoto Y, Hirata T, Abe Y, Takayama M, Maeno T (2022). Association between multimorbidity, self-rated health and life satisfaction among independent, community-dwelling very old persons in Japan: longitudinal cohort analysis from the Kawasaki ageing and well-being project. BMJ Open.

[22] Sato Y, Atarashi K, Damian R, Arai Y, Sasajima S, Sean MK (2021). Novel bile acid biosynthetic pathways are enriched in the microbiome of centenarians. Nature.

[23] Tao Y, Oguma Y, Asakura K, Abe Y, Arai Y. Association between dietary patterns and subjective and objective measures of physical activity among japanese adults aged 85 years and older: a cross-sectional study. Br J Nutr. 2022;1–10. 10.1017/S0007114522003993 10.1017/S0007114522003993 PMC10442796 36573371

[24] Arai Y, Oguma Y, Abe Y, Takayama M, Hara A, Urushihara H, Takebayashi T (2021). Behavioral changes and hygiene practices of older adults in Japan during the first wave of COVID-19 emergency. BMC Geriatr.

[25] Kobayashi S, Yuan X, Sasaki S, Osawa Y, Hirata T, Abe Y (2019). Relative validity of brief-type self-administered diet history questionnaire among very old japanese aged 80 years or older. Public Health Nutr.

[26] Ministry of Health, Labour, and Welfare (2017). The National Health and Nutrition Survey in Japan, 2015.

[27] Sasaki S, Yanagibori R, Amano K (1998). Self-administered diet history questionnaire developed for health education: a relative validation of the test-version by comparison with 3-day diet record in women. J Epidemiol.

[28] Satomi K, Kentaro M, Satoshi S, Hitomi O, Naoko H, Akiko N (2011). Comparison of relative validity of food group intakes estimated by comprehensive and brief-type self-administered diet history questionnaires against 16 d dietary records in japanese adults. Public Health Nutr.

[29] The Tokyo Oldest Old Survey on Total Health (TOOTH) (2010). A longitudinal cohort study of multidimensional components of health and well-being. BMC Geriatr.

[30] Hirata T, Arai Y, Yuasa S, Abe Y, Takayama M, Sasaki T et al. Nat Commun. Associations of cardiovascular biomarkers and plasma albumin with exceptional survival to the highest ages. Nat Commun. 2020;11:3820. 10.1038/s41467-020-17636-0 10.1038/s41467-020-17636-0 PMC7393489 32732919

[31] Mahoney FI, Barthel DW (1965). Functional evaluation: the Barthel index. Md State Med J.

[32] Lawton MP, Brody EM (1969). Assessment of older people: self-maintaining and instrumental activities of daily living. Gerontologist.

[33] Folstein MF, Folstein SE, McHugh PR (1975). Mini-mental state’. A practical method for grading the cognitive state of patients for the clinician. J Psychiatr Res.

[34] Arai H, Ministry of Health LaW (2019). Guide to prevent long term care (in japanese). Subsidy for promotion of health care services for the elderly in 2019 (Health promotion services for the elderly).

[35] David C. Modelling Survival Data in Medical Research. 4th ed. Chapman and Hall/CRC. 2023. p. 60–62.

[36] Dietary Intake Standards for Japanese. (2020 Edition) “Dietary Intake Standards for Japanese” Formulation Study Group Report p133. https://www.mhlw.go.jp/content/10904750/000586553.pdf. Accessed 20 Mar 2023.

[37] Pedersen AN, Kondrup J, Børsheim E (2013). Health effects of protein intake in healthy adults: a systematic literature review. Food Nutr Res.

[38] Pilz S, Tomaschitz A, Drechsler C, Zittermann A, Dekker JM, März W (2011). Vitamin D supplementation: a promising approach for the prevention and treatment of strokes. Curr Drug Targets.

[39] He K, Merchant A, Rimm EB, Rosner BA, Stampfer MJ, Willett WC, Ascherio A (2004). Folate, vitamin B6, and B12 intakes in relation to risk of stroke among men. Stroke.

[40] Militante JD, Lombardini JB (2002). Treatment of hypertension with oral taurine: experimental and clinical studies. Amino Acids.

[41] Moncada S, Higgs A (1993). The L-arginine-nitric oxide pathway. N Engl J Med.

[42] D’Elia L, Barba G, Cappuccio FP, Strazzullo P (2011). Potassium intake, stroke, and cardiovascular disease a meta-analysis of prospective studies. J Am Coll Cardiol.

[43] Hoption Cann SA (2006). Hypothesis: dietary iodine intake in the etiology of cardiovascular disease. J Am Coll Nutr.

[44] Serhan CN (2014). Pro-resolving lipid mediators are leads for resolution physiology. Nature.

[45] Shirota T, Haji S, Yamasaki M, Iwasaki T, Hidaka T, Takeyama Y (2005). Apoptosis in human pancreatic cancer cells induced by eicosapentaenoic acid. Nutrition.

[46] Cao W, Ma Z, Rasenick MM, Yeh SY, Yu JZ (2012). N-3 poly-unsaturated fatty acids shift estrogen signaling to inhibit human breast cancer cell growth. PLoS ONE.

[47] Calviello G, Di Nicuolo F, Gragnoli S, Piccioni E, Serini S, Maggiano N (2004). n-3 PUFAs reduce VEGF expression in human colon cancer cells modulating the COX-2/PGE2 induced ERK-1 and – 2 and HIF-1alpha induction pathway. Carcinogenesis.

[48] Lee CY-K, Sit W-H, Fan S-T, Man K, Jor IW-Y, Wong LL-Y (2010). The cell cycle effects of docosahexaenoic acid on human metastatic hepatocellular carcinoma proliferation. Int J Oncol.

[49] Eser PO, Vanden Heuvel JP, Araujo J, Thompson JT (2013). Marine- and plant-derived ω-3 fatty acids differentially regulate prostate cancer cell proliferation. Mol Clin Oncol.

[50] Yao QH, Zhang X-C, Fu T, Gu JZ, Wang L, Wang Y (2014). ω-3 polyunsaturated fatty acids inhibit the proliferation of the lung adenocarcinoma cell line A549 in vitro. Mol Med Rep.

[51] Yokoyama M, Origasa H, Matsuzaki M, Matsuzawa Y, Saito Y, Ishikawa Y (2007). Effects of eicosapentaenoic acid on major coronary events in hypercholesterolaemic patients (JELIS): a randomised open-label, blinded endpoint analysis. Lancet.

[52] Zhuang P, Zhang Y, He W, Chen X, Chen J, He L (2019). Dietary Fats in Relation to Total and cause-specific mortality in a prospective cohort of 521 120 individuals with 16 years of Follow-Up. Circ Res.

[53] Watanabe M, Higashiyama A, Kokubo Y, Ono Y, Okayama A, Okamura T, Nippon, DATA80/90 Research Group (2010). Protein intakes and serum albumin levels in a japanese general population: NIPPON DATA90. J Epidemiol.

[54] Phillips A, Shaper AG, Whincup PH (1989). Association between serum albumin and mortality from cardiovascular disease, cancer, and other causes. Lancet.

[55] Djoussé L, Rothman KJ, Cupples LA, Levy D, Ellison RC (2002). Serum albumin and risk of myocardial infarction and all-cause mortality in the Framingham offspring study. Circulation.

[56] Schalk BW, Visser M, Bremmer MA, Penninx BW, Bouter LM, Deeg DJ (2006). Change of serum albumin and risk of cardiovascular disease and all-cause mortality: Longitudinal Aging Study Amsterdam. Am J Epidemiol.

